# Aberrant MEK5/ERK5 signalling contributes to human colon cancer progression via NF-*κ*B activation

**DOI:** 10.1038/cddis.2015.83

**Published:** 2015-04-09

**Authors:** A E S Simões, D M Pereira, S E Gomes, H Brito, T Carvalho, A French, R E Castro, C J Steer, S N Thibodeau, C M P Rodrigues, P M Borralho

**Affiliations:** 1 Research Institute for Medicines (iMed.ULisboa), Faculty of Pharmacy, Universidade de Lisboa, Av. Prof. Gama Pinto, Lisbon 1649-003, Portugal; 2 Histology and Comparative Pathology Laboratory, Instituto de Medicina Molecular, Av. Prof. Egas Moniz, Edificio Egas Moniz, Lisbon 1649-028, Portugal; 3 Department of Laboratory Medicine and Pathology, Mayo Clinic, 200 First Street S.W., Rochester, MN 55905, USA; 4 Department of Medicine, VFW Cancer Research Center, University of Minnesota Medical School, 406 Harvard Street S.E., Minneapolis, MN 55455, USA; 5 Department of Genetics, Cell Biology and Development, VFW Cancer Research Center, University of Minnesota Medical School, 406 Harvard Street S.E., Minneapolis, MN 55455, USA

## Abstract

This study was designed to evaluate MEK5 and ERK5 expression in colon cancer progression and to ascertain the relevance of MEK5/ERK5 signalling in colon cancer. Expression of MEK5 and ERK5 was evaluated in 323 human colon cancer samples. To evaluate the role of MEK5/ERK5 signalling in colon cancer, we developed a stable cell line model with differential MEK5/ERK5 activation. Impact of differential MEK5/ERK5 signalling was evaluated on cell cycle progression by flow cytometry and cell migration was evaluated by wound healing and transwell migration assays. Finally, we used an orthotopic xenograft mouse model of colon cancer to assess tumour growth and progression. Our results demonstrated that MEK5 and ERK5 are overexpressed in human adenomas (*P*<0.01) and adenocarcinomas (*P*<0.05), where increased ERK5 expression correlated with the acquisition of more invasive and metastatic potential (*P*<0.05). Interestingly, we observed a significant correlation between ERK5 expression and NF-*κ*B activation in human adenocarcinomas (*P*<0.001). We also showed that ERK5 overactivation significantly accelerated cell cycle progression (*P*<0.05) and increased cell migration (*P*<0.01). Furthermore, cells with overactivated ERK5 displayed increased NF-*κ*B nuclear translocation and transcriptional activity (*P*<0.05), together with increased expression of the mesenchymal marker vimentin (*P*<0.05). We further demonstrated that increased NF-*κ*B activation was associated with increased I*κ*B phosphorylation and degradation (*P*<0.05). Finally, in the mouse model, lymph node metastasis was exclusively seen in orthotopically implanted tumours with overactivated MEK5/ERK5, and not in tumours with inhibited MEK5/ERK5. Our results suggested that MEK5/ERK5/NF-*κ*B signalling pathway is important for tumour onset, progression and metastasis, possibly representing a novel relevant therapeutic target in colon cancer treatment.

Colon cancer (CC) remains the third most commonly diagnosed non-cutaneous cancer in both sexes.^1^ Despite significant improvements achieved in CC treatment, metastatic CC remains almost incurable.^2, 3^ Therefore, understanding CC molecular circuitry to further identify new druggable therapeutic targets is highly desirable to improve therapeutic efficacy and outcome in CC.

Interestingly, MAPK signalling pathways have been shown to be widely deregulated in cancer. MAPK family consist of four major MAPK subfamilies, including extracellular-regulated protein kinases 1/2 (ERK1/2), c-Jun N-terminal kinase, p38 MAPKs and ERK5.^4^ ERK5 activation is generally prompted by mitogens and cellular stress, and proceeds by sequential activation of MEKK2/3 and MEK5.^5^ The activation of ERK5 generally triggers the downstream activation of several transcription factors and other effectors that regulate multiple crucial cellular events, including proliferation, survival and apoptosis.^5, 6, 7^ Importantly, aberrant ERK5 signalling has already been reported in several different cancers, including breast,^8^ prostate,^9^ oral squamous cell,^10^ hepatocellular^11^ and T-cell leukemia,^12^ and is generally associated with poorer prognosis. ERK5 appears to be a critical embryonic factor for angiogenesis and has been shown to be essential for angiogenesis in melanoma and lung carcinoma xenografts,^13^ and for prostate cancer metastasis.^14^

MAPK signalling pathway is a downstream target of Ras family members and activating KRAS mutations are one of the main features underlying CC development and resistance to therapy.^15^ Importantly, activation of MEK5 has recently been associated with decreased overall survival of CC patients.^16^ Collectively, MEK5/ERK5 overexpression or increased activation in several human cancers and its association with a poorer disease survival makes ERK5 a potential desirable target for the development of additional novel cancer therapies. Furthermore, there is growing evidence to suggest an interaction between ERK5 and NF-*κ*B signalling pathways. Importantly, NF-*κ*B signalling pathway is commonly overly activated in several types of cancer, including CC, and is considered a promising therapeutic target.^17^ Several studies indicated that ERK5-dependent NF-*κ*B activation is important for survival in mitosis, cell cycle progression and tumour xenograft growth.^6, 12, 18, 19^

Collectively, exploring the ERK5 and NF-*κ*B signalling axis is extremely relevant in CC, where ERK5 may be a novel therapeutic target. Therefore, the aims of the present study were to evaluate the steady-state expression profiles of MEK5 and ERK5, and NF-*κ*B and I*κ*B in 323 well-characterized human colon adenomas and adenocarcinomas, and also determine the potential biological role of MEK5/ERK5 signalling in CC. Collectively, the present study highlights the relevance of the therapeutic targeting of MEK5/ERK5 signalling as a promising therapeutic strategy in CC.

## Results

### MEK5/ERK5 and NF-*κ*B signalling pathways are deregulated in human colon adenomas and adenocarcinomas

**Figure 1:**
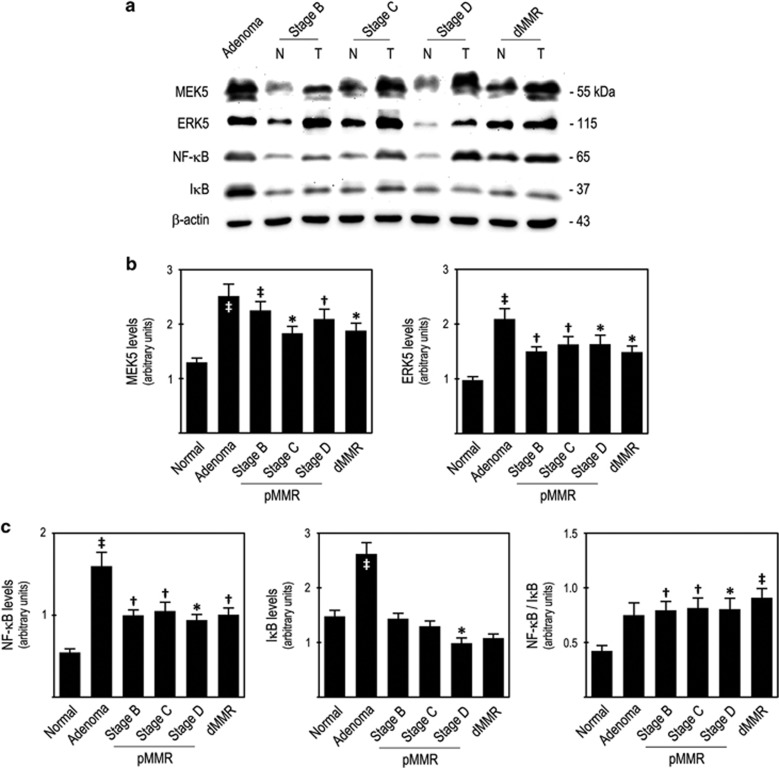

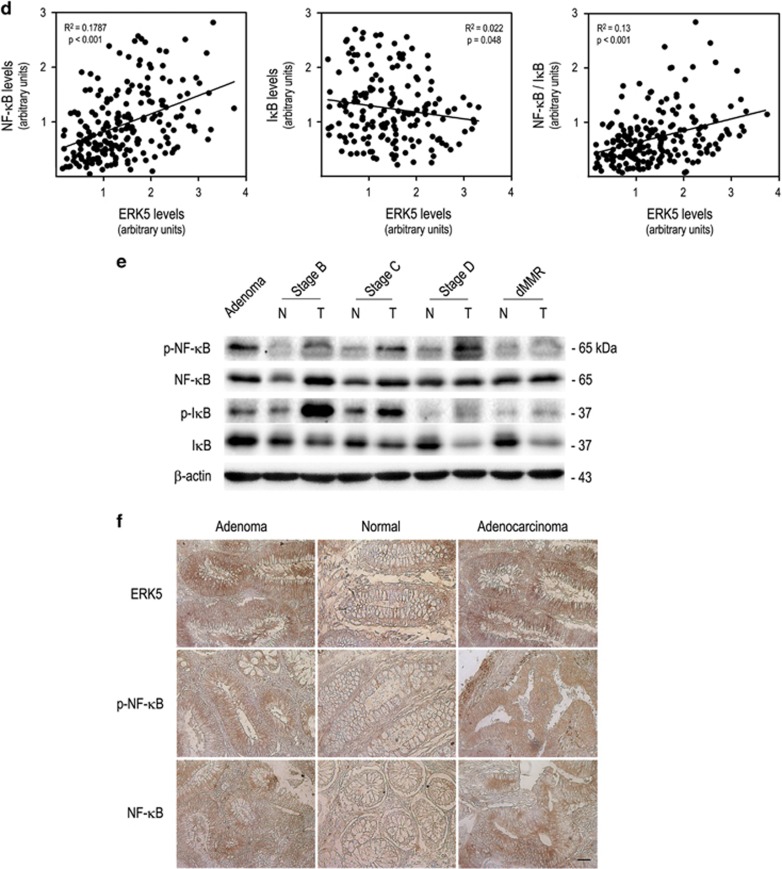
MEK5/ERK5 and NF-*κ*B signalling pathways are deregulated in human CC, with ERK5 expression correlating with increased NF-*κ*B activation. (**a**) Representative immunoblots of steady-state protein expression levels of MEK5, ERK5, NF-*κ*B, I*κ*B and *β*-actin in normal colon, colon adenomas and pMMR and dMMR colon carcinomas; (**b**) MEK5 and ERK5 steady-state protein levels; (**c**) NF-*κ*B, I*κ*B and NF-*κ*B/I*κ*B ratio; (**d**) correlations between ERK5 steady-state protein levels and NF-*κ*B, I*κ*B or NF-*κ*B/I*κ*B ratio; (**e**) representative immunoblots of steady-state levels of p-NF-*κ*B, NF-*κ*B, p-I*κ*B, I*κ*B and *β*-actin in normal colon, colon adenoma, and pMMR and dMMR colon carcinomas; and (**f**) representative immunohistochemistry for ERK5, p-NF-*κ*B and NF-*κ*B in human colon cancer samples. Immunoblot statistical significance was determined using the non-parametric statistical analysis Kruskal–Wallis test with Dunn's post test for selected comparisons and results are expressed as mean±S.E.M. for samples in each category; correlation statistical significance was determined using the non-parametric stastistical analysis Spearman test. pMMR, proficient mismatch repair system; dMMR, deficient mismatch repair system. Scale bar=100 *μ*m. *P<0.05, ^†^*P*<0.01 and ^‡^*P*<0.001 from normal colon

To evaluate the significance of MEK5/ERK5 signalling in human CC, we examined the steady-state levels of MEK5 and ERK5 proteins in our set of 323 human CC samples. Our results clearly showed that MEK5 steady-state levels were significantly increased in human colon adenomas (*P*<0.001), stage B–D proficient DNA mismatch repair (pMMR) carcinomas (stage B, *P*<0.01; stage C, *P*<0.05; stage D, *P*<0.01) and in defective MMR (dMMR) carcinomas (*P*<0.05), compared with normal colon mucosa (Figures 1a and b, left panel). In addition, ERK5 steady-state levels were also significantly increased in human colon adenomas (*P*<0.001), in stage B–D pMMR carcinomas (stage B, *P*<0.01; stage C, *P*<0.01; stage D, *P*<0.05) and also in dMMR carcinomas (*P*<0.05), compared with normal colonic mucosa (Figures 1a and b, right panel, and Figure 1f).

In parallel, we evaluated the steady-state levels of NF-*κ*B and its inhibitor I*κ*B, and also NF-*κ*B/I*κ*B ratio as an indirect estimate of NF-*κ*B activation. Importantly, we observed a deregulation of NF-*κ*B signalling pathway in our set of human CC samples, with increased NF-*κ*B steady-state levels in human colon adenomas (*P*<0.001), stage B–D pMMR carcinomas (stage B and C, *P*<0.01; stage D, *P*<0.05) and dMMR carcinomas (*P*<0.01), compared with normal colon mucosa (Figure 1c, left panel, and 1f). Interestingly, I*κ*B steady-state levels were significantly increased in adenomas (*P*<0.001) and decreased in stage D pMMR carcinomas (*P*<0.05), compared with normal colon mucosa (Figure 1c, middle panel). In turn, NF-*κ*B/I*κ*B ratios showed that NF-*κ*B activity was increased in adenomas, although not reaching statistical significance. NF-*κ*B activity was found to be significantly increased in pMMR (stage B and C, *P*<0.01; stage D, *P*<0.05) and dMMR (*P*<0.001) carcinomas compared with normal colon mucosa (Figure 1c, right panel). To further ascertain the activation of NF-*κ*B signalling pathway in these human samples, we also determined p-NF-*κ*B and p-I*κ*B steady-state levels, verifying their increase in adenoma and adenocarcinoma samples in comparison with normal colon mucosa (Figures 1e and f).

Our results confirmed that deregulation of NF-*κ*B signalling pathway in CC may accompany, or result from, deregulation of the ERK5 signalling pathway. Consequently, we tested the potential correlation between ERK5 steady-state levels with that of NF-*κ*B, I*κ*B and ultimately with NF-*κ*B activation, in each CC sample. We observed a significant positive correlation between the protein steady-state levels of ERK5 and NF-*κ*B (*P*<0.001), and ERK5 and NF-*κ*B activation (*P*<0.001), and a significant negative correlation between ERK5 and I*κ*B protein steady-state levels (*P*<0.05; Figure 1d).

Collectively, our data suggested the existence of interplay between ERK5 and NF-*κ*B signalling pathways that might be biologically relevant for CC onset and progression.

### Increased ERK5 steady-state expression and NF-*κ*B activation are correlated with tumour progression

We next ascertained whether MEK5, ERK5, NF-*κ*B and I*κ*B steady-state levels and NF-*κ*B activation in human colon adenocarcinoma samples were correlated with tumour clinicopathological characteristics. Interestingly, our results demonstrated that increased ERK5 steady-state levels and NF-*κ*B activation were significantly correlated with increased depth of invasion (*P*<0.05) and with the presence of lymph node metastasis (*P*<0.05) and distant metastasis (*P*<0.01), and no significant association was seen for gender, age at presentation or with tumour location (Table 1). In addition, we performed a similar analysis for I*κ*B and observed that decreased levels of I*κ*B were correlated with invasion (*P*<0.01), lymph node (*P*<0.05) and distant metastasis (*P*<0.01; Table 1). No significant correlation was observed between NF-*κ*B or MEK5 steady-state expression levels and clinicopathological data (Table 1). Importantly, the results obtained demonstrated that increased ERK5 steady-state levels and NF-*κ*B activation were significantly further increased in tumours presenting more invasive phenotypes (T_4_) in comparison with less invasive tumours (T_1–3_; *P*<0.05). In addition, ERK5 expression and NF-*κ*B activation are also significantly further increased in tumours with more than four regional lymph nodes involved (N2) and in cases with distant metastasis at presentation (M1), compared with tumours with less aggressive phenotypes (*P*<0.05 *versus* N0–1 and M0, respectively; Table 1). Interestingly, our results suggested that increased NF-*κ*B activation may result primarily from decreased I*κ*B steady-state levels rather than from increased NF-*κ*B expression.

### MEK5/ERK5 activation promotes cell cycle progression through NF-*κ*B activation

**Figure 2:**
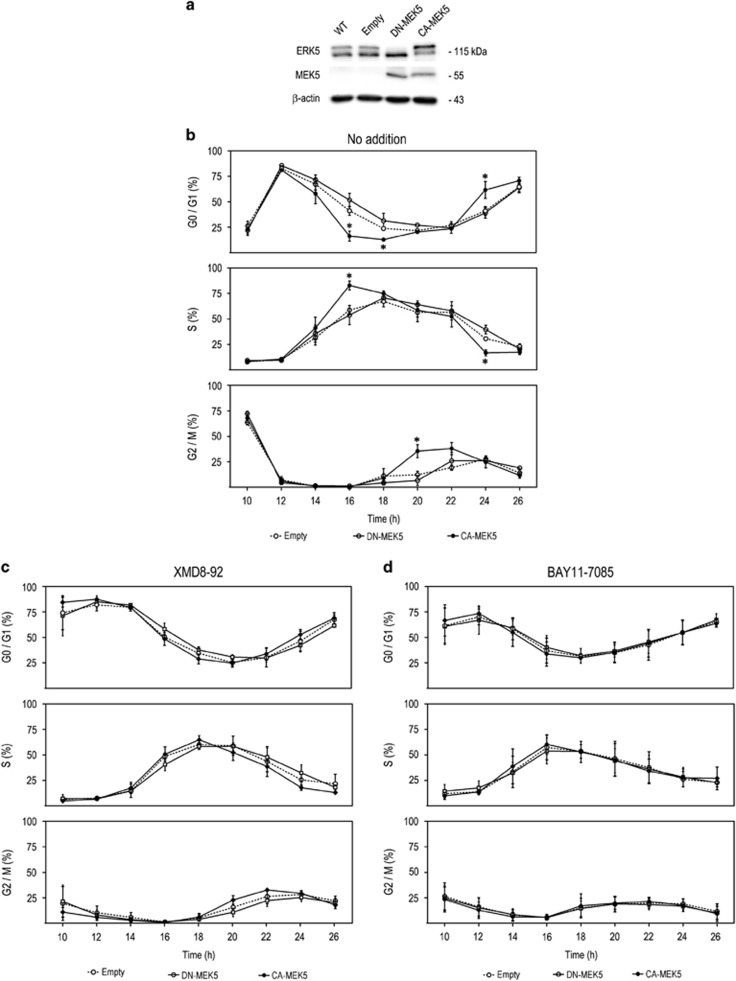
MEK5/ERK5 activation accelerates cell cycle progression in SW620 cells. (**a**) Representative immunoblot of ERK5 protein levels in SW620 cell lines with differential ERK5 activation, stable constitutive MEK5 activation (*CA-MEK5*), dominant-negative MEK5 (*DN-MEK5*), empty control (*Empty*) and wild-type SW620 cell line (WT). The developed cell model consistently showed that *DN-MEK5* led to constitutive inhibition of ERK5 activation, *CA-MEK5* led to constitutive ERK5 activation and *Empty* control cells displayed basal ERK5 activation. (**b**) FACS cell cycle analysis of CA-MEK5, DN-MEK5 and Empty SW620 cells, following release from dual-thymidine block and exposed to (**c**) XMD8-92 or (**d**) BAY11-7085. Significance was determined using ANOVA test with Tukey's post test for selected comparisons and results are expressed as mean±S.E.M. from at least three independent experiments. *P<0.05 from *Empty* cell line

To evaluate the impact of MEK5/ERK5 signalling pathway on human CC, we established SW620-derived cell lines with differential MEK5/ERK5 activation, namely: constitutive MEK5 activation (*CA-MEK5*), dominant-negative MEK5 (*DN-MEK5*) and empty vector (*Empty*) control (Figure 2a). Further, we evaluated the role of differential MEK5/ERK5 activation in cell cycle progression in G1–S synchronized cells. Our results demonstrated that *CA-MEK5* cells displayed significantly accelerated cell cycle progression (Figure 2b). In particular, at 16 h after release from G1–S block, 83% of *CA-MEK5* cells progressed into S-phase, whereas only 55% of *DN-MEK5* and *Empty* cells entered S phase (*P*<0.05). Next, at 20 h 38% of *CA-MEK5* cells had already transitioned to G2/M phase, in contrast with *DN-MEK5* and *Empty* cells, which were only starting to enter G2/M (*P*<0.05). By 24 h, 60% of *CA-MEK5* cells were in G1 phase compared with <45% of *DN-MEK5* and *Empty* cells (*P*<0.05; Figure 2b). In order to ascertain the involvement of ERK5 and/or NF-*κ*B in the increased cell cycle progression resulting from MEK5 activation, we exposed our cell model to XMD8-92 or BAY11-7085 (ERK5 and NF-*κ*B inhibitors, respectively) and evaluated cell cycle progression. Interestingly, our results demonstrated that the increased cell cycle progression was completely abolished after inhibition of ERK5 or NF-*κ*B (Figures 2c and d, respectively).

**Figure 3:**
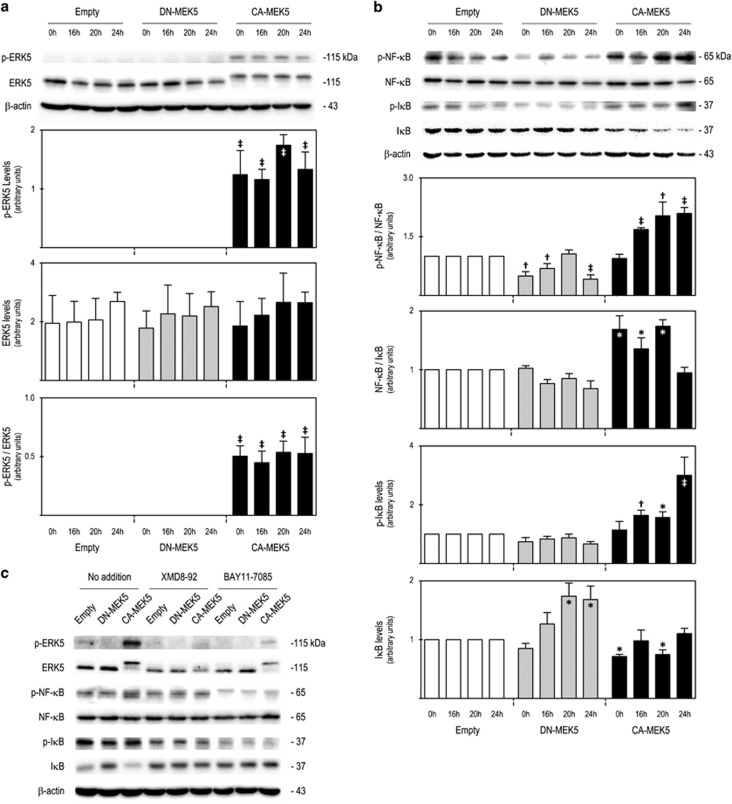
MEK5/ERK5 activation accelerated cell cycle is associated with NF-*κ*B activation via reduction of I*κ*B steady-state levels. Immunoblot analysis of steady-state protein levels of (**a**) p-ERK5, ERK5 and p-ERK5/ERK5, and of (**b**) p-NF-*κ*B/NF-*κ*B, NF-*κ*B/I*κ*B ratios, p-I*κ*B and I*κ*B. (**c**) Representative immunoblots for p-ERK5, ERK5, p-NF-*κ*B, NF-*κ*B, p-I*κ*B, I*κ*B and *β*-actin in Empty, DN-MEK5 and CA-MEK5 SW620 cells when treated for 24 h with XMD8-92 and BAY11-7085, or untreated (no addition). Significance was determined using ANOVA test with Tukey's post test for selected comparisons and results are expressed as mean±S.E.M. from at least three independent experiments. *P<0.05, ^†^*P*<0.01 and ^‡^*P*<0.001 from *Empty* cell line

We confirmed ERK5 constitutive activation was found only in *CA-MEK5* cells, whereas no significant alterations were found in ERK5 steady-state levels between the model cell lines (Figure 3a). In contrast, the NF-*κ*B signalling pathway was significantly modulated between cell lines during cell cycle progression with a significant increase in p-NF-*κ*B/NF-*κ*B and NF-*κ*B/I*κ*B ratio in *CA-MEK5* cells (*P*<0.05) and a decrease of p-NF-*κ*B/NF-*κ*B ratio in *DN-MEK5* cells (*P*<0.05), and a trend decrease of NF-*κ*B/I*κ*B ratio (Figure 3b). Further, we observed that after treatment with ERK5 inhibitor, the increased steady-state levels of p-NF-*κ*B presented in *CA-MEK5* cells was reduced to levels similar to those presented by Empty and DN-MEK5 cells (Figure 3c). Collectively, our results indicate that MEK5/ERK5 activation promotes cell cycle progression via NF-*κ*B activation.

### MEK5/ERK5 activation increases human CC cell migration through NF-*κ*B activation

**Figure 4:**
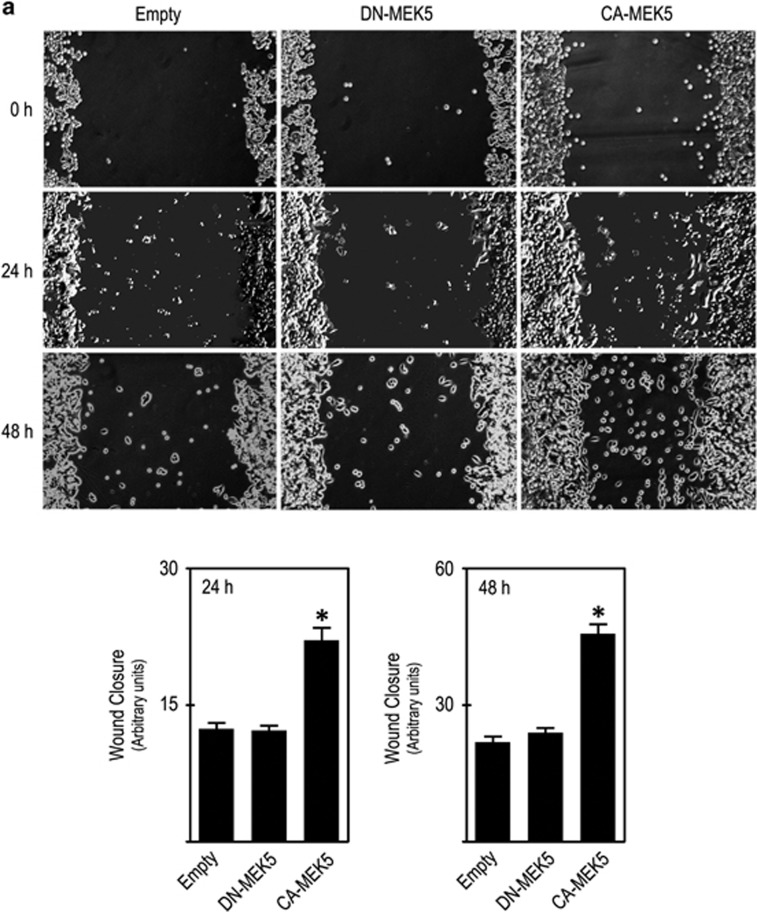

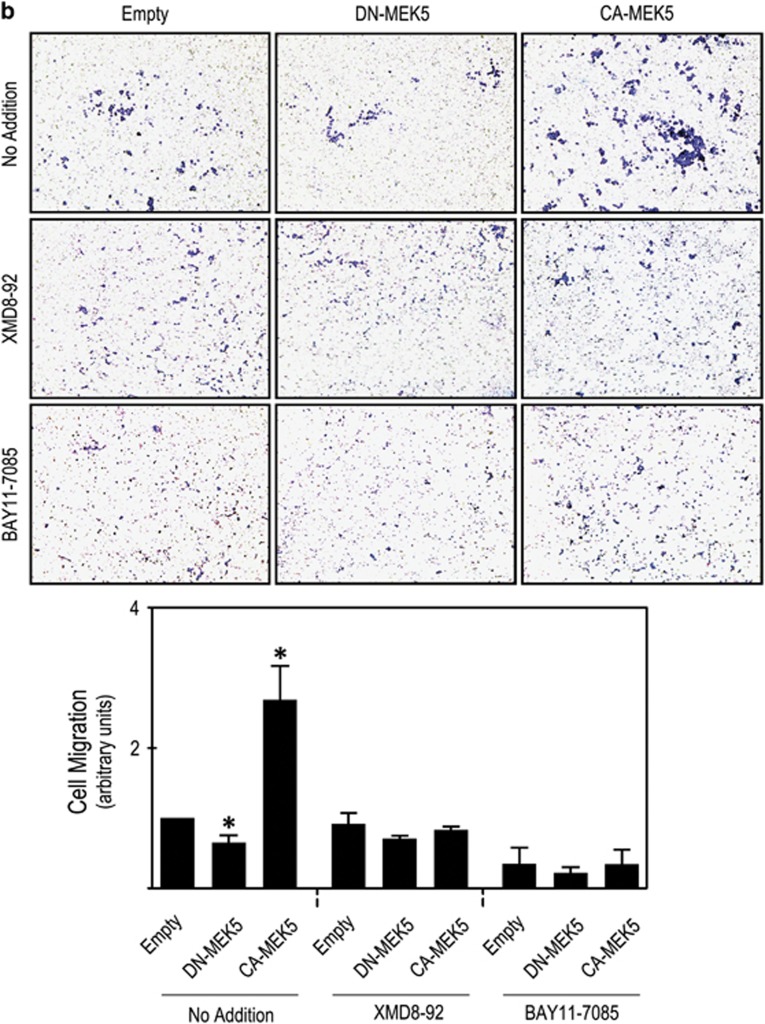
MEK5/ERK5 activation increases cell migration *in vitro*. Cell migration was assessed by (**a**) wound-healing assay at 24 and 48 h after ‘wound' formation and (**b**) transwell migration assay, with cells allowed to migrate for 9 h after cell platting. Significance was determined using ANOVA test with Tukey's post test for selected comparisons and results are expressed as mean±S.E.M. from at least three independent experiments. *P<0.05 from *Empty* cell line

We next evaluated the role of MEK5/ERK5 activation in CC cells. Wound-healing assay demonstrated that *CA-MEK5* cells migrated nearly twofold more than *DN-MEK5* and *Empty* cells, both at 24 or 48 h (*P*<0.001; Figure 4a). In addition, transwell migration showed that *CA-MEK5* cells displayed a threefold increase in migration through 8 *μ*m polycarbonate membrane filter compared with *DN-MEK5* and *Empty* cells (*P*<0.05; Figure 4b, upper panel). Therefore, our results demonstrated that MEK5/ERK5 activation promoted human CC cell migration. In agreement with our cell cycle data, increased migration of CA-MEK5 cells was abrogated by incubation with ERK5 or NF-*κ*B inhibitors (Figure 4b, middle and lower panels).

**Figure 5:**
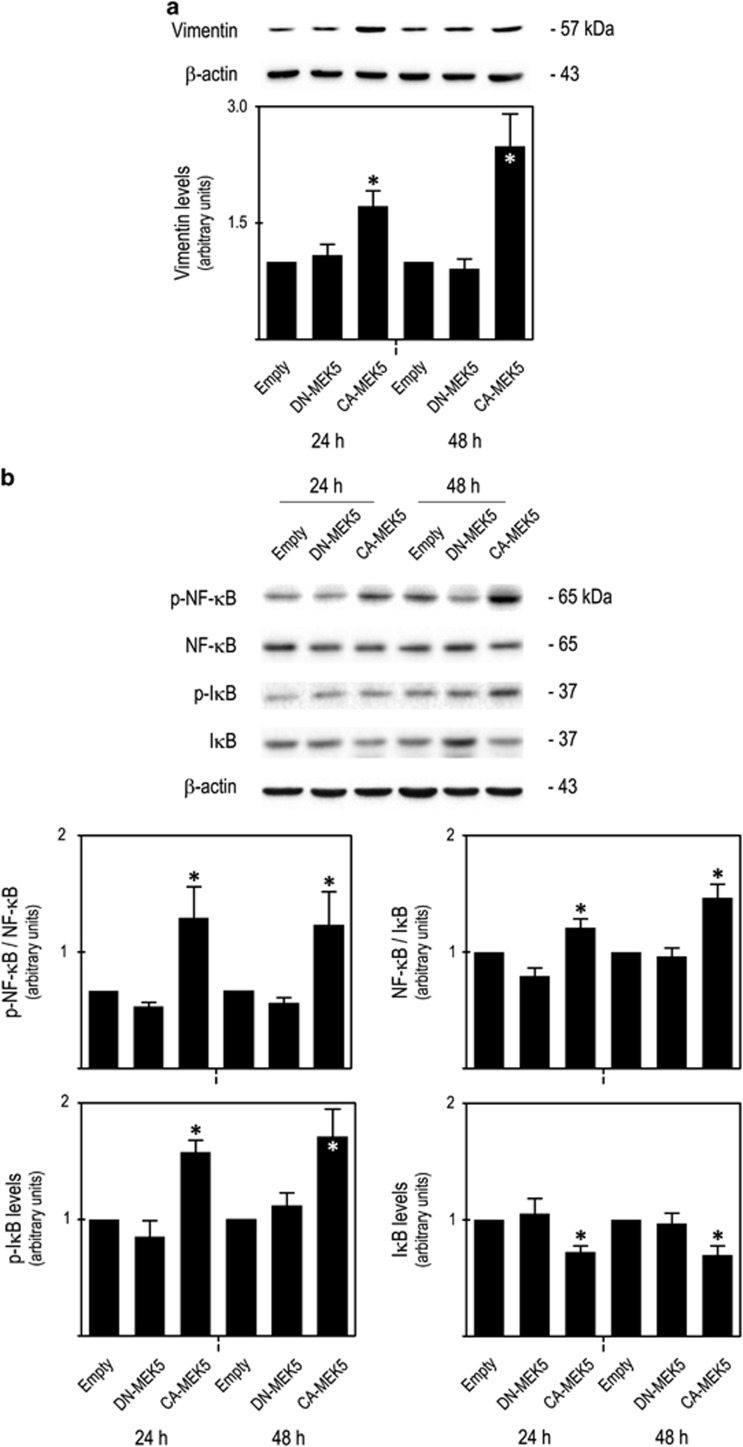
MEK5/ERK5 activation increased cell migration is associated with increased vimentin expression and NF-*κ*B activation. Immunoblot analysis of steady-state protein levels of (**a**) vimentin and (**b**) of p-NF-*κ*B/NF-*κ*B, NF-*κ*B/I*κ*B ratios, p-I*κ*B and I*κ*B. Significance was determined using ANOVA test with Tukey's post test for selected comparisons and results are expressed as mean±S.E.M. of at least three independent experiments. *P<0.05 from *Empty* cell line

Further, we observed that increased migration of *CA-MEK5* cells was accompanied by an increase of Vimentin steady-state levels (Figure 5a), a crucial regulator of cell migration and epithelial-to-mesenchymal transition, and also by an increase of p-NF-*κ*B/NF-*κ*B and NF-*κ*B/I*κ*B ratios (*P*<0.05), resulting from a significant increase in p-I*κ*B protein steady-state levels and reduction of total I*κ*B protein steady-state levels (*P*<0.05; Figure 5b). No significant differences were observed regarding NF-*κ*B protein levels (data not shown). Interestingly, NF-*κ*B has already been reported to directly regulate Vimentin expression by binding to its promoter, thus contributing to cancer progression.^20, 21^ Therefore, the increased steady-state levels of Vimentin in *CA-MEK5* cells might have resulted from increased NF-kB activation. Taken together, our results suggested that MEK5/ERK5 signalling promotes NF-*κ*B activation and Vimentin expression, which contribute to increased tumour cell migration.

### MEK5/ERK5 signalling induces NF-*κ*B activation via I*κ*B degradation

**Figure 6:**
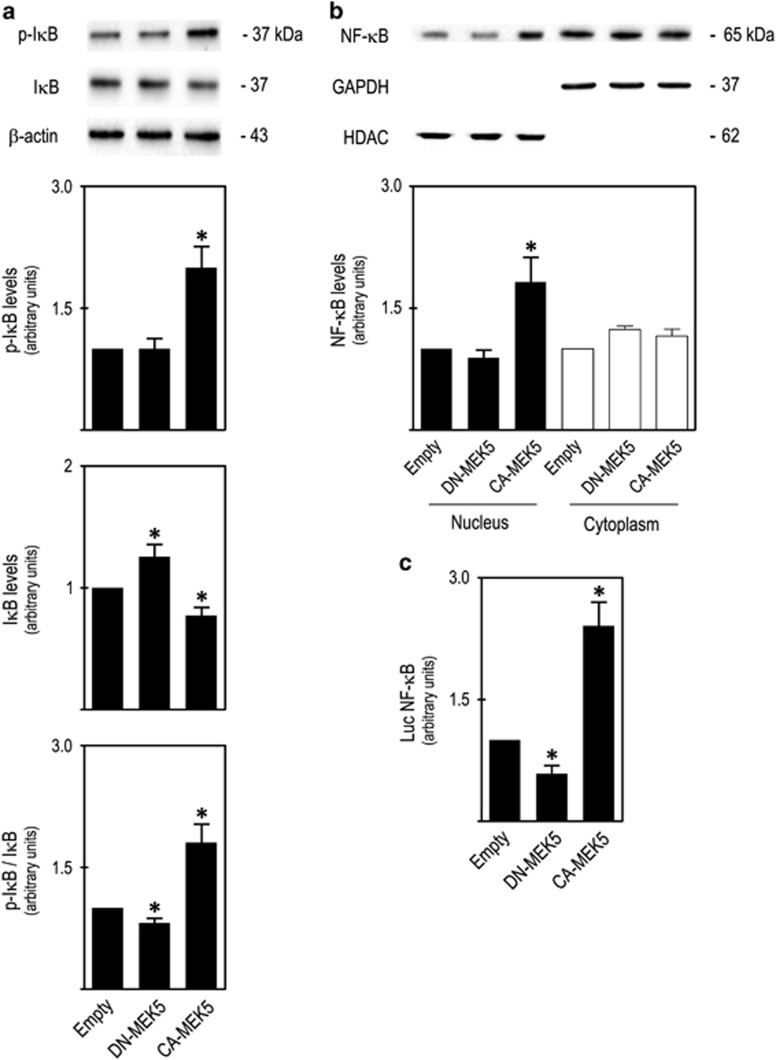
MEK5/ERK5 activation increases NF-*κ*B nuclear translocation and transcriptional activity via I*κ*B phosphorylation and degradation. Immunoblot analysis of steady-state protein levels of **(a)** p-I*κ*B, I*κ*B and p-I*κ*B/I*κ*B ratios, and (**b**) nuclear and cytosolic NF-*κ*B; (**c**) NF-*κ*B transcriptional activity, evaluated by dual luciferase assay with reporter plasmids. Significance was determined using ANOVA test with Tukey's post test for selected comparisons and results are expressed as mean±S.E.M. from at least three independent experiments. *P<0.05 from *Empty* cell line

ERK5 is reported to function as an IKKα kinase,^19^ promoting I*κ*B degradation and consequently NF-*κ*B release from I*κ*B. Our results suggested an interaction between ERK5 and NF-*κ*B signalling pathways, in which ERK5 promotes NF-*κ*B activation via I*κ*B degradation and consequent release of NF-*κ*B from I*κ*B (Figures 1,3 and 5). Therefore, to test this hypothesis we evaluated I*κ*B phosphorylation, NF-*κ*B nuclear translocation and NF-*κ*B transcriptional activity in our SW620 cell model. Our results showed a significant increase in I*κ*B phosphorylation in *CA-MEK5* cells compared with *DN-MEK5* and *Empty* cells (*P*<0.05; Figure 6a), together with a significant increase in NF-*κ*B nuclear translocation (*P*<0.05; Figure 6b). Using NF-*κ*B transcriptional activity reporter plasmids, we confirmed that *CA-MEK5* cells displayed significantly higher NF-*κ*B transcriptional activity in comparison with *DN-MEK5* and *Empty* cells (*P*<0.05; Figure 6c). Interestingly, we also observed a significant decrease in NF-*κ*B transcriptional activity in *DN-MEK5* cells in comparison with *Empty* cells (*P*<0.05; Figure 6c). Taken together, our results demonstrated that MEK5/ERK5 signalling leads to NF-*κ*B activation, in part via I*κ*B degradation.

### MEK5/ERK5 signalling promotes *in vivo* human CC metastasis

**Figure 7:**
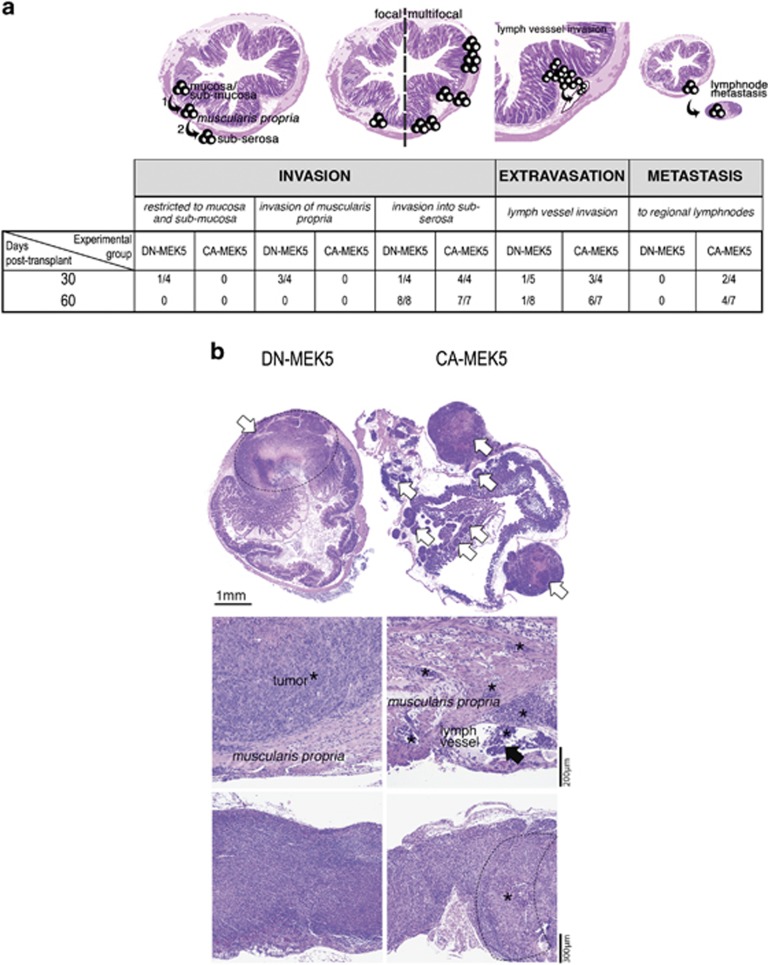
*In vivo*, MEK5/ERK5 activation is associated with local invasion and regional lymphnode metastasis. As shown in the graphical abstract, (**a**) the injected tumours may grow locally in the caecum/colon infiltrating mucosa, sub-mucosa, *muscularis propria* and eventually sub-serosa. In addition, tumour growth may be focal (restricted to one foci, at the injection site) or multifocal (numerous foci spread throughout the caecum and colon, not restricted to the injection site). Furthermore, in addition to local invasion by tumour cells, the metastatic cascade encompasses intravasation into lymph vessels, extravasation out of the circulation, and survival and growth at secondary site. We injected 5 × 10^5^ SW620 DN-MEK5 or CA-MEK5 cells into the wall of the caecum, in BALB/c *scid* mice, and mice were killed 30 or 60 days post injection. (**a**) Histopathological characteristics of DN-MEK5 and CA-MEK5 tumours regarding local invasion, extravasation and distant metastasis (to regional lymphnodes), and (**b)** representative microphotographs of the multifocallity of CA-MEK5 tumours, compared with the focal lesions seen in DN-MEK5 tumours (white arrows, upper panel), of the lymph vessel invasion (black arrowhead, middle panel) and of the lymphnode metastasis (lower panel). *Tumour cells

To further evaluate the role of MEK5/ERK5 signalling in CC cell invasion and metastasis *in vivo*, implantation of CA-MEK5 or DN-MEK5 cells into the cecum wall of BALB/c *scid* mice was performed. Our data showed that 30 days post injection, CA-MEK5 tumours were more invasive, with more extravasation and with lymph node metastasis (in 50% of the mice; Figure 7a). Further, at day 60 days post injection, although all DN-MEK5 tumours were also seen to infiltrate into sub-serosa, these tumours were mostly focal (~88%) compared with CA-MEK5 tumours that were multifocal in over 70% of the mice (Figure 7b, upper panels). Furthermore, DN-MEK5 tumours rarely showed extravasation and failed to develop lymph node metastasis, compared with 85% of the CA-MEK5 tumours that showed extravasation and >50% were spread to regional lymph nodes (Figure 7a). Collectively, our results demonstrate that MEK5/ERK5 activation is relevant for CC cell infiltrative and metastatic potential.

## Discussion

The relevance of MEK5/ERK5 signalling in cancer is increasing exponentially due to its demonstrated pro-survival, pro-proliferative and pro-angiogenic roles.^5, 6, 7^ Further, aberrant expression of MEK5/ERK5 was already reported in several human cancers.^8, 9, 10, 11, 14, 22, 23^ Our results now demonstrate the overexpression of MEK5 and ERK5 in human colon adenomas and adenocarcinomas. More importantly, the early overexpression of ERK5 in adenomas suggests that ERK5 may be a key factor in the transition from normal colon to adenoma. This is consistent with the downregulation of miR-143, one of the main regulators of ERK5 expression, which has also been reported to occur in the transition of normal colon to adenoma,^24, 25^ and may be responsible for the increased ERK5 steady-state levels. Interestingly, ERK5 steady-state levels are not significantly altered between dMMR and pMMR tumours, suggesting that ERK5 overexpression is independent of MMR system status, therefore representing a common event in CC tumorigenesis. Our data supports the addition of CC to the growing list of cancer types displaying aberrant MEK5/ERK5 expression, thus increasing the relevance of these kinases as putative novel therapeutic targets in cancer.

The role of NF-*κ*B signalling pathway is now well established in carcinogenesis. NF-*κ*B overexpression and constitutive activation has already been reported in several tumour types, including CC, lymphoma, leukemia, breast cancer, pancreatic adenocarcinoma and gastric carcinoma.^26^ In addition, tumours with increased NF-*κ*B activity present higher resistance to treatment.^27, 28^ In the present work, we confirmed the aberrant expression and activation of NF-*κ*B in CC. Importantly, we found a significant correlation between ERK5 steady-state levels and NF-*κ*B signalling, where higher levels of ERK5 were associated with increased levels of NF-*κ*B, reduced expression of I*κ*B and increased NF-*κ*B activation. ERK5 has been shown to activate NF-*κ*B, inducing nuclear translocation, where it is involved in mitosis, and G2–M cell cycle progression.^6, 12, 18, 19^ Although inhibition of NF-*κ*B has been considered as a potential therapeutic target in cancer, inhibitors of this pathway are unlikely to be practical agents, as they can lead to severe immunodeficiency.^17^ Our data would suggest a significant correlation between ERK5 expression and NF-*κ*B signalling pathway deregulation in human CC patients, and a potential synergism between ERK5 and NF-*κ*B signalling pathways in the pathogenesis and progression of CC.

Aberrant ERK5 signalling is increasingly associated with more aggressive phenotypes and a poorer disease prognosis in several tumour types. In this regard, higher levels of ERK5 expression were associated with metastasis to bone and a poorer prognosis in prostate cancer.^14^ In oral squamous cell carcinoma, higher levels of p-ERK5 expression were correlated with more advanced tumour stage and the presence of lymph node involvement.^10^ In breast cancer, MEK5 expression via STAT3 was shown to provide crucial survival signals to tumour cells,^29^ and conferred chemoresistance.^30^ Importantly, in CC, p-MEK5 expression levels were shown to correlate with increased invasion and metastasis, and with a decrease in overall survival.^16^ In the present study, we report that ERK5 steady-state levels are increased in colon adenomas and adenocarcinomas, and that ERK5 expression status was significantly correlated with CC progression. We observed that ERK5 is highly expressed in T_4_ carcinomas and, to a lesser extent in T_1–3_ carcinomas. Similarly, we observed that N_2_ and M_1_ carcinomas presented higher ERK5 levels when compared with less aggressive carcinomas, N_0–1_ and M_0_, respectively. These results demonstrated that ERK5 expression is positively correlated with tumour-node-metastasis (TNM) stage, and that higher levels of ERK5 are associated with more advanced cancer stages. In agreement with previous observations in which higher levels of p-MEK5 were correlated with T_3–4_, N_1–2_ and M_1_ carcinomas, our results suggest that ERK5 signalling pathway could potentially be used as a biomarker, or as a prognostic factor for colon cancer progression.^16^

NF-*κ*B signalling pathway influences cancer via a number of different mechanisms. Indeed, it has been reported that constitutive activation of NF-*κ*B represents a common event in several human tumours, including CC. Its sustained activation during inflammation may increase the potential neoplastic transformation of normal cells.^17, 31^ One proposed mechanism implicates defective I*κ*B*α* activity and the resulting failure to sequester NF-*κ*B in the cytosol.^31^ Here we confirmed the increased activation of NF-*κ*B in human colon tumours was correlated with tumour progression. In fact, higher levels of NF-*κ*B activation were observed in T_4_, N_2_ and M_1_ carcinomas, in comparison with the less aggressive carcinomas, T_1–3_, N_0–1_ and M_0_ carcinomas. Somewhat curiously, we observed that increased NF-*κ*B activation was not due to increased NF-*κ*B expression, but rather due to decreased I*κ*B steady-state levels in T_4_, N_2_ and M_1_ carcinomas.

Using a CC SW620 cell model with differential MEK5/ERK5 activation status, we demonstrated that MEK5/ERK5 activation significantly promoted cell cycle progression. In this regard, it has already been demonstrated that ERK5 was essential to sustain cell proliferation by promoting G1–S phase transition of cell cycle.^32^ ERK5 activation was also shown to be essential in G2/M cell cycle transition.^6^ Although several reports demonstrated that ERK5 has a role in cellular proliferation, the underlying mechanisms involved in cell cycle and particularly at the G1–S transition are not well defined and, in some cases, remain controversial. For example, the inhibition of ERK5 activation promoted cell cycle arrest at the G1–S phase in NIH3T3 cells.^33^ ERK5 activation was shown to also reduce cell cycle arrest^34^ and to activate cyclin D1 expression.^35^ In contrast, fibroblasts derived from MEK5^−/−^ mice did not present defects on G1–S phase transition,^36^ although these animals experienced early embryonic death. Here we demonstrated that ERK5 activation might be important for both G1–S and S–G2/M phase transitions.

ERK5 has been associated with angiogenesis, migration and tumour metastasis.^13, 14, 37^ We demonstrated that MEK5/ERK5 activation *in vitro* significantly contributed to CC cell migration, and that orthotopically implanted CC cells with constitutive MEK5/ERK5 activation develop tumours with increased invasive and metastatic potential (to regional lymphnodes). In contrast, *in vitro* inhibition of MEK5/ERK5 activity was significantly associated with lower migration ability of CC cells and, *in vivo*, inhibited MEK5/ERK5 activity failed to develop lymph node metastasis.

The ability for tumour cells to migrate is generally associated with an overall worse clinical prognosis. Indeed, tumour cells usually undergo several morphological and molecular alterations that ultimately result in increasingly resistant and aggressive tumours. Here we observed that *CA-MEK5* cells presented a significant overexpression of the mesenchymal marker Vimentin. Although Vimentin expression is generally associated with epithelial-to-mesenchymal transition, the participation of vimentin in several tumorigenic events has already been reported.^38, 39^ Vimentin activates RAS-MEK-ERK signalling, promotes cell migration and invasion,^40, 41^ inhibits differentiation^42^ and has recently been suggested to neutralize the activity of natural killer cells.^39^

Another very important aspect regarding cancer onset and progression is the interplay between tumorigenic signalling pathways. In this regard, we observed that accelerated G1–S and S–G2/M cell cycle transition and increased tumour cell migration in *CA-MEK5* cells were accompanied by a significant increase of NF-*κ*B activation, along with a decrease in the total levels of I*κ*B. Together with the results from our patient data, where I*κ*B expression was inversely correlated with ERK5 expression, our data suggested that ERK5 activation promotes NF-*κ*B activation, possibly through I*κ*B degradation. In fact, previous studies have demonstrated that NF-*κ*B inhibition through ERK5 inhibition diminishes cell proliferation and increases cell apoptosis.^12, 18^ Our own results demonstrated that ERK5 activation significantly increases I*κ*B phosphorylation, leading to decreased total levels of I*κ*B and consequently to increased NF-*κ*B nuclear translocation and higher NF-*κ*B transcriptional activity. More interestingly, ERK5 inhibition was associated with a significant decrease in NF-*κ*B transcriptional activity compared with control. These results support the potential beneficial role of ERK5 signalling pathway inhibition in CC therapy.

In conclusion, our study demonstrated aberrant expression of ERK5 and MEK5 in human CC adenomas and adenocarcinomas, where increased ERK5 levels correlated with cancer progression and spread. Further, we also demonstrated that ERK5 activation, mediated by MEK5, promoted NF-*κ*B activation via increased targeting of I*κ*B to degradation. In addition, our results strongly suggested that aberrant ERK5 signalling and NF-*κ*B activation contributes to increased tumour cell proliferation, migration and metastasis, and that this pathway may be a potential novel, and extremely relevant therapeutic target in human CC.

## Materials and Methods

### Human colon cancer tissue

Human colon specimens were obtained from previous studies, where total RNA and protein were extracted to evaluate microRNA expression profiles^43^ and steady-state protein expression in CC,^44^ respectively. Normal areas of colonic epithelium were obtained from either the margin of resection or adjacent to the tumour. Rectal cancers were excluded. Polyps were evaluated for histologic type, with only tubulovillous adenomas selected for study. Pathologic tumour staging was carried out using Dukes staging and TNM system.^45, 46^ MMR status was evaluated as previously described.^43^ Further, all samples included in the present study were anonymized and comprised 323 colon samples, including 53 normal colon, 42 adenomas, 72 dMMR carcinomas and 156 pMMR carcinomas (Table 1).

The Mayo Clinic Institutional Review Board reviewed and approved for human studies the protocol entitled, ‘The Identification and Validation of miRNA Signature Profiles as Biomarkers for Colon Cancer Progression' from Dr Stephen N Thibodeau. The Committee noted that the human study aspects involved the use of samples collected under IRB-approved protocols. The Committee determined that the consenting process allows for future use of the samples as exemplified in the current protocol. The majority of patients provided written informed consent and samples were anonymized for those who did not.

### Immunohistochemistry

Three-micrometre-thick, paraffin-embedded human colon sections were deparaffined, rehydrated and boiled three times in 10 mM citrate buffer, pH 6, for epitope retrieval. Next, endogenous peroxidase was quenched by incubating sections in 3% hydrogen peroxide in PBS for 5 min at room temperature (RT). Subsequently, sections were incubated for 1 h in blocking buffer (10% (v/v) normal donkey serum (Jackson ImmunoResearch Laboratories, Inc., West Grove, PA, USA) in PBS containing 0.4% (v/v) Triton X-100 (Sigma-Aldrich, St. Louis, MO, USA)). Next, sections were incubated overnight at 4 °C with primary rabbit antibody reactive to ERK5 (#3372, 1 : 50; Cell Signalling, Beverly, MA, USA), primary rabbit antibody reactive to p-NF-*κ*B (#ab131109, 1 : 50; Abcam, Cambridge, UK) and primary mouse antibody reactive to NF-*κ*B (sc-8008, 1 : 50; Santa Cruz Biotechnology, Inc., Santa Cruz, CA, USA). After rinsing in PBS, the detection of primary antibodies was performed using HiDef Detection HRP Polymer System (Cell Marque, Rocklin, CA, USA), according to the manufacturer's instructions. In brief, sections were incubated with HiDef Detection Amplifier for 10 min at RT, rinsed and incubated with HiDef Detection HRP Polymer Detector for 10 min at RT. After rinsing, peroxidase activity was detected using SIGMAFAST 3,3′-Diaminobenzidine tablets (Sigma) at RT. Finally, slides were rinsed, dehydrated and a glass coverslip was mounted using Fluoromount-GTM mounting media (Beckman Coulter, Inc., Fullerton, CA, USA). The specimens were examined using a bright-field microscope using a Axio Scope A.1 fluorescence microscope (Zeiss Axioskop; Carl Zeiss GmbH, Jena, Germany). Images were acquired, under a × 400 magnification, using a DFC490 camera (Leica Microsystems AG, Heersbrugg, Switzerland) with the IM50 software for image acquisition (Leica Microsystems, version 1.20, Release 9).

### Cell lines and plasmids

pWPI-GFP lentiviral expression constructs encoding constitutively active MEK5 (pWPI-MEK5DD), dominant-negative MEK5 (pWPI-MEK5AA) and empty vector control (pWPI), with simultaneous transgene and eGFP expression, were kindly provided by Dr Robert C Doebele (University of Colorado, CO, USA).^47^ Lentiviral particles were packaged in HEK293T cells, using packaging plasmids pGag-pol and pRev, and envelope plasmid pVSV-G. Briefly, HEK293T cells were co-transfected with (3 : 2 : 1 : 4) of pGag-pol : pRev : pVSV-G : lentiviral expression vector pWPI-MEK5DD/pWPI-MEK5AA/pWPI, using Lipofectamine 2000, according to the manufacturer's instructions (Invitrogen, Life Technologies, Grand Island, NY, USA). Lentiviral particle-containing supernatants were collected 48 and 72 h after transfection, pooled and filtered using a 0.45-*μ*m filter and stored at −80 °C, until use.

### Generation of SW620 colon adenocarcinoma cells with differential MEK5 activation

SW620 cells with differential activation of MEK5/ERK5 were produced by stable overexpression of constitutively active MEK5 (*CA-MEK5*), dominant-negative MEK5 (*DN-MEK5*) and empty vector (*Empty*), by transduction of SW620 cells with pWPI-MEK5DD, pWPI-MEK5AA and pWPI viral particles, respectively. *CA-MEK5*, *DN-MEK5* and *Empty* stable cell lines were purified by cell sorting of GFP-expressing cells, obtaining over 90% GFP-positive cells, and this remained unchanged for all the experiments.

### Cell culture

SW620 and HEK293T cell lines were cultured in DMEM supplemented with 10% fetal bovine serum (FBS) and 1% antibiotic/antimycotic solution (all from Gibco, Life Technologies, Paisley, UK) and were maintained at 37 °C in a humidified atmosphere of 5% CO_2_.

### Cell cycle analysis

Cells were synchronized at G1/S phase transition by double thymidine block, as previously described.^48^ Subsequently, at the indicated timepoints, cells were collected for FACS and western blot analysis. Briefly, synchronized cells were centrifuged and ressuspended in ice-cold PBS and fixed under gentle vortexing by dropwise addition of an equal volume of ice-cold 80% ethanol (−20 °C), followed by 30 min on ice. Subsequently, samples were stored at 4 °C for at least 18 h, followed by incubation with RNase A solution (10 *μ*g/ml, in PBS) for 30 min at 37 °C. Finally, propidium iodide (25 *μ*g/ml) was added to the samples for 5 min, before flow cytometry analysis. In selected experiments, cells were incubated with ERK5 inhibitor XMD8-92 (#4132, TOCRIS Bioscience, Bristol, UK) or NF-*κ*B inhibitor BAY11-7085 (#sc-202490, from Santa Cruz Biotechnology, Inc.) at 4 *μ*M, or vehicle (DMSO), when cells were subjected to the second thymidine block, and the presence of inhibitors was maintained until the indicated endpoints.

### Wound-healing assay

Cells were grown to confluence in 30 mm^2^ dishes and ‘wounds' were performed using a 200-*μ*l sterile pipette tip. Cells were allowed to close the ‘wound' for the indicated time periods and ‘wound' images were captured with a Zeiss Primo Vert microscope (Carl Zeiss Microscopy GmbH, Jena, Germany) connected to a Leica DFC 40 camera (Leica Microsystems AG). The total empty ‘wound' area (cell free) was measured using Image J software (http://rsbweb.nih.gov/ij/). ‘Wound' closure was calculated by subtracting the ‘wound' area at the indicated time periods from the initial ‘wound' area.

### Transwell migration chamber assay

Cells were seeded in six-well plates and 24 h later were serum starved for 16 h, by replacing complete media with serum-free medium with our without XMD8-92 (4 *μ*M) or BAY11-7085 (4 *μ*M). Subsequently, serum-starved cells were replated in Neuro Probe 48-well Micro Chemotaxis Chamber (Neuro Probe, Inc, Gaithersburg, MD, USA). To determine migration, 5 × 10^4^ cells were seeded in the upper chamber in serum-free medium on top of a polycarbonate membrane filter with 8 *μ*m pore (Neuro Probe, Inc). Cells were allowed to migrate for 9 h across the polycarbonate membrane filter, to the lower chamber containing complete medium (10% FBS) as chemotractant. In cells exposed to XMD8-92 or BAY11-7085, the inhibitors were present in the upper and lower chamber throughout the migration assay. Subsequently, non-migrated cells at the membrane top were removed and migrated cells were fixed with cold methanol followed by Giemsa staining. Number of migrated cells per well was determined by counting the total cells per well, using image J software, and images were captured by bright-field microscopy.

### Total, nuclear and cytosolic protein isolation

Total protein extracts were prepared from CC tissues and cell lines. Frozen CC tissue ∼7 mm^2^ and 10 *μ*m thick were sectioned and placed in 400 *μ*l of RLT buffer (QIAGEN, Chatsworth, CA, USA) including 4 *μ*l of *β*-mercaptoethanol. Samples were stored at −80 °C until use for RNA extraction using TRIzol LS (Invitrogen), according to the manufacturer's instructions.^43^ Total protein isolation from organic phases of TRizol-chloroforms tissue samples was performed as previously described by us.^44^ For total protein isolation from cell lines, cells were collected and processed as previously described by us.^49^ To evaluate nuclear translocation of NF-*κ*B, nuclear and cytosolic extracts were prepared as previously described.^50^

### Immunoblotting

The steady-state levels of ERK5, p-ERK5, MEK5, NF-*κ*B, p-NF-*κ*B, I*κ*B-*α*, p-I*κ*B-*α*, Vimentin, *β*-actin, HDAC and GAPDH were determined by immunoblot analysis, as previously described.^44^ Briefly, blots were incubated overnight at 4 °C with primary rabbit antibody reactive to ERK5 (#3372), p-ERK5 (#3371) or primary mouse antibody reactive to p-I*κ*B-*α* (#9246; all from Cell Signalling) or primary rabbit antibody reactive to NF-*κ*B (#sc-372), I*κ*B-*α* (#sc-371) or primary mouse antibody reactive to MEK5 (#sc-135986), Vimentin (#sc-32322), GAPDH (#sc-32233), *β*-actin (#sc-8432; all from Santa Cruz Biotechnology) or HDAC (#05-614, Merck Millipore Corp, Billerica, MA, USA) or primary rabbit antibody reactive to p-NF-*κ*B (#ab131109; Abcam). Next, immunoblots were incubated with anti-rabbit or -mouse secondary antibody conjugated with horseradish peroxidase (Bio-Rad Laboratories, Hercules, CA, USA) for 3 h at RT. The relative intensifies of protein bands were analysed using the densitometric analysis software Image Lab version 5.1—beta, using a Chemidoc MP Imaging System for acquisition (Bio-Rad Laboratories).

### NF-*κ*B transcriptional activity reporter assay

NF-*κ*B transcriptional activity was measured using Cignal NF-*κ*B Pathway Reporter Assay Kit (QIAGEN), following the manufacturer's protocol. Firefly and renilla activities were measured using the Dual-Luciferase Reporter Assay System (Promega Corporation, Madison, WI, USA). Renilla luciferase activity was used as a transfection normalization control.

### Animal model and histopathology

All experimental procedures were performed according to EU recommendations for good practices and animal welfare, and approved by the IMM Animal Care and Ethical Committee (AEC_2014_08_PB_Cancer). An othotopic xenograft mouse model of colon cancer was established to assess the role of differential MEK5 activation on primary tumour growth and metastasis. Briefly, 8- to 12-week-old male BALB/c *scid* mice were anaesthetized and midline laparotomy was performed; the caecum was exposed and 5 × 10^5^ cells were injected in the visceral surface of the caecal wall. The caecum was then rinsed with PBS and gently placed inside the abdominal cavity, followed by closure of the abdominal wall and skin. Analgesia was performed, animals were monitored daily and every effort was made to minimize suffering. Mice were killed with CO_2_ narcosis 30 or 60 days post transplantation, necropsy was performed and selected organs (caecum, colon, small intestine, mesenteric lymph node, liver, spleen, lung and heart) were collected, fixed in 10% neutral-buffered formalin and embedded in paraffin. Next, 3-*μ*m sections were stained with haematoxylin and eosin for routine histopathology. Tissue sections were examined by a pathologist, blinded to experimental groups, in a Leica DM2500 microscope coupled to a Leica MC170 HD microscope camera.

### Statistical analysis

All data are expressed as mean±S.E.M. from at least three independent experiments. Statistical analysis was performed using GraphPad Prism 5.00 software (GraphPad Software Inc., San Diego, CA, USA). Regarding immunoblot analysis in Figure 1, significance was determined using the non-parametric Kruskal–Wallis test with Dunn's post test for selected comparisons, whereas for correlations analysis significance was determined using the non-parametric Spearman test (patient data). ANOVA test with Tukey's post test was used for selected comparisons when more than two groups were analysed and Student's *t*-test when two groups were analysed. Values of *P*<0.05 were considered statistically significant.

## Figures and Tables

**Table 1:** Correlation between MEK5, ERK5, NF-*κ*B and I*κ*B steady-state protein expression, NF-*κ*B activation and clinicopathological characteristics

**Variable**	**Cases**	**MEK5 (S.E.M.)**	***P*-value**	**ERK5 (S.E.M.)**	***P*-value**	**NF-κB (S.E.M.)**	***P*-value**	**IκB (S.E.M.)**	***P*-value**	**NF-κB/IκB (S.E.M.)**	***P*-value**
Gender			0.457		0.905		0.966		0.731		0.922
Male	147	2.26 (0.14)		1.55 (0.11)		1.09 (0.06)		1.53 (0.10)		0.81 (0.22)	
Female	125	2.42 (0.15)		1.53 (0.09)		1.09 (0.07)		1.58 (0.11)		0.80 (0.23)	
Age at surgery, years			0.993		0.747		0.450		0.440		0.720
40–49	2	1.60 (0.79)		0.91 (0.40)		1.33 (0.92)		1.57 (0.62)		0.62 (0.15)	
50–59	45	1.82 (0.10)		1.41 (0.10)		1.25 (0.14)		1.77 (0.19)		0.75 (0.07)	
60–69	86	1.84 (0.11)		1.36 (0.08)		1.07 (0.07)		1.54 (0.13)		0.67 (0.05)	
70–79	88	1.81 (0.10)		1.42 (0.09)		1.05 (0.09)		1.63 (0.14)		0.74 (0.05)	
80–89	46	1.85 (0.13)		1.46 (0.11)		0.99 (0.08)		1.35 (0.16)		0.83 (0.09)	
>90	5	1.32 (0.17)		1.77 (1.39)		1.66 (0.58)		0.79 (0.14)		0.59 (0.17)	
Tumour location			0.217		0.321		0.409		0.651		0.873
Caecum	67	1.75 (0.10)		1.37 (0.11)		0.98 (0.09)		1.44 (0.14)		0.70 (0.05)	
Ascending colon	59	1.80 (0.11)		1.59 (0.13)		1.09 (0.10)		1.50 (0.14)		0.84 (0.08)	
Hepatic flexure	9	1.36 (0.11)		1.39 (0.10)		0.93 (0.14)		1.47 (0.28)		0.59 (0.10)	
Anonymous right side	3	1.45 (0.94)		2.19 (1.39)		0.88 (0.56)		1.43 (0.10)		0.59 (0.35)	
Transverse colon	29	1.80 (0.18)		1.53 (0.13)		1.31 (0.19)		1.26 (0.17)		0.74 (0.08)	
Splenic flexure	9	1.72 (0.23)		1.50 (0.08)		1.17 (0.29)		1.94 (0.41)		0.65 (0.13)	
Descending colon	17	2.30 (0.35)		1.06 (0.16)		1.43 (0.21)		1.85 (0.37)		0.70 (0.10)	
Sigmoid colon	72	1.94 (0.12)		1.57 (0.09)		1.03 (0.09)		1.71 (0.18)		0.74 (0.08)	
Anonymous left side	7	1.66 (0.10)		1.28 (0.22)		1.28 (0.33)		1.91 (0.60)		0.73 (0.34)	
pT (invasion depth)			0.276		**0.047**		0.827		**0.007**		**0.030**
T_1_	5	1.80 (0.32)		1.35 (0.05)		0.98 (0.36)		1.88 (0.42)		0.54 (0.07)	
T_2_	53	1.85 (0.10)		1.29 (0.11)		0.85 (0.08)		1.35 (0.11)		0.80 (0.10)	
T_3_	156	1.90 (0.07)		1.42 (0.05)		0.90 (0.04)		1.18 (0.05)		0.77 (0.05)	
T_4_	19	1.55 (0.26)		2.18 (0.41)^a^		0.80 (0.18)		0.71 (0.10)^a^		1.04 (0.16)^a^	
pN (lymph node metastasis)			0.590		**0.023**		0.608		**0.030**		**0.024**
N_0_	132	1.90 (0.06)		1.32 (0.05)		0.90 (0.05)		1.26 (0.06)		0.73 (0.04)	
N_1_	69	1.83 (0.10)		1.43 (0.10)		0.91 (0.08)		1.12 (0.08)		0.77 (0.07)	
N_2_	32	1.75 (0.13)		1.73 (0.12)^b^		1.01 (0.09)		0.97 (0.09)^b^		0.93 (0.10)^b^	
pM (distant metastasis)			0.169		**0.006**		0.409		**0.005**		**0.023**
M_0_	195	1.78 (0.06)		1.35 (0.05)		0.91 (0.05)		1.31 (0.07)		0.75 (0.04)	
M_1_	38	1.90 (0.14)		1.52 (0.14)		0.93 (0.08)		0.91 (0.08)		0.91 (0.10)	

pT and pN significance was determined using Kruskal–Wallis test with Dunn's post test for selected comparisons and pM significance was determine using Student's *t*- test

a Statistically significant in comparison with T_1_, T_2_ and T_3_

b Statistically significant in comparison with N_0_

## Abbreviations

BALB/c *Scid*: BALB mice carrying the severe combined immune deficiency mutation
CA-MEK5: constitutive active mitogen-activated protein kinase kinase 5
CC: colon cancer
DN-MEK5: dominant negative mitogen-activated protein kinase kinase 5
ERK5: extracellular signal-regulated kinase 5
FACS: fluorescent automated cell sorting
I*κ*B: nuclear factor of kappa light polypeptide gene enhancer in B-cells inhibitor
IKK: I*κ*B (nuclear factor of kappa light polypeptide gene enhancer in B-cells inhibitor) kinase
KRAS: Kirsten rat sarcoma viral oncogene homolog
MAPK: mitogen-activated protein kinase
MEK5: mitogen-activated protein kinase kinase 5
MEKK2/3: mitogen-activated protein kinase kinase kinase 2/3
MMR: mismatch repair system
NF-*κ*B: nuclear factor of kappa light polypeptide gene enhancer in B-cells
TNM: tumor node metastasis
